# Improved Resin–Zirconia Bonding by Room Temperature Hydrofluoric Acid Etching

**DOI:** 10.3390/ma8030850

**Published:** 2015-03-02

**Authors:** Mun-Hwan Lee, Jun Sik Son, Kyo-Han Kim, Tae-Yub Kwon

**Affiliations:** 1 Department of Medical & Biological Engineering, Graduate School, Kyungpook National University, 2-188-1 Samduk-dong, Jung-gu, Daegu 700-412, Korea; E-Mail: leemunhwan@knu.ac.kr; 2 Korea Textile Development Institute, 1083 Jungri-dong, Seo-gu, Daegu 703-712, Korea; E-Mail: sonjk1@empas.com; 3 Department of Dental Biomaterials, School of Dentistry, Kyungpook National University, 2-188-1 Samduk-dong, Jung-gu, Daegu 700-412, Korea; E-Mail: kyohan@knu.ac.kr

**Keywords:** zirconia ceramic, resin–zirconia bonding, etching, hydrofluoric acid

## Abstract

This *in vitro* study was conducted to evaluate the shear bond strength of “non-self-adhesive” resin to dental zirconia etched with hydrofluoric acid (HF) at room temperature and to compare it to that of air-abraded zirconia. Sintered zirconia plates were air-abraded (control) or etched with 10%, 20%, or 30% HF for either 5 or 30 min. After cleaning, the surfaces were characterized using various analytical techniques. Three resin cylinders (Duo-Link) were bonded to each treated plate. All bonded specimens were stored in water at 37 °C for 24 h, and then half of them were additionally thermocycled 5000 times prior to the shear bond-strength tests (*n* = 12). The formation of micro- and nano-porosities on the etched surfaces increased with increasing concentration and application time of the HF solution. The surface wettability of zirconia also increased with increasing surface roughness. Higher concentrations and longer application times of the HF solution produced higher bond-strength values. Infiltration of the resin into the micro- and nano-porosities was observed by scanning electron microscopy. This *in vitro* study suggests that HF slowly etches zirconia ceramic surfaces at room temperature, thereby improving the resin–zirconia bond strength by the formation of retentive sites.

## 1. Introduction

Today, yttria-stabilized polycrystalline tetragonal zirconia (Y-TZP) ceramic is one of the most commonly used all-ceramic core dental materials, mainly because of its superior strength and high fracture toughness [1,2]. Zirconia itself is rather hydrophobic and has a low surface free energy [3]. Its very low (approximately 5%) surface concentration of –OH groups suggests that only a very small number of reactive groups are available for chemical bonding [4,5]. Thus, the non-reactive surface of zirconia produces a consistent issue of concern, namely low adhesion potential to other substrates [5].

Bonding to traditional silica-based ceramics is a predictable procedure yielding durable results if certain guidelines are followed [6]. The standard protocol involves etching of the silica-based ceramic surfaces with hydrofluoric acid (HF) and coating with a silane-coupling agent [7]. The HF etching process forms a microretentive surface with high free energy that increases the interaction between the ceramic and the resin cement [6,7].

However, it has been reported that zirconia ceramic is not readily etched by HF owing to its high crystal1inity, making it difficult to roughen the surface for mechanical retention [2,8,9,10,11,12]. On the other hand, air-abrasion using Al_2_O_3_ particles has been found to be effective in cleaning and roughening zirconia surfaces [10,13,14]. Wegner and Kern demonstrated that a durable resin–zirconia bonding can be achieved by air-abrasion in combination with resin materials containing an adhesive monomer, such as 10-methacryloyloxydecyl dihydrogenphosphate (MDP) [15]. Novel surface-roughening techniques for zirconia have also been explored, and selective infiltration etching is one of them. It uses a heat-induced maturation process to pre-stress the surface grain boundaries in zirconia in order to allow infiltration of molten glass into these boundaries [9]. The glass is then etched out using HF, thereby creating a 3D network of intergranular pores that allows nanomechanical interlocking of resin cement [9,16,17]. Another experimental method is to use a hot chemical etching solution although no research on the bond strength of zirconia to resin cements has been reported using this treatment [9]. Furthermore, such a procedure is hazardous and practically difficult, especially at high temperatures [18].

The reliability and long-term durability of bonding between zirconia and resin is determined by micromechanical and chemical retention [11,12]. With regard to chemical retention, MDP-containing resin cements or primers seem to be most appropriate because of the chemical interaction between the hydroxyl groups of the passive zirconia surface and the phosphate ester group of MDP [12]. Nonetheless, adequate micromechanical retention to zirconia would still be advantageous because the chemical bonding does not provide long-term bond strength without sufficient micromechanical retention [10,14]. It has also been demonstrated that conventional bisphenol A diglycidyl methacrylate (Bis-GMA)-based resins without adhesive monomers are unable to create long-term durable bonds to zirconia [12,14,15,19]. However, it was assumed that HF etching of zirconia creates different surface topology than that obtained by air-abrasion, thereby resulting in enhanced resin–zirconia bond strength, even without the use of MDP monomer-containing resins or primers.

The purpose of this *in vitro* study was to test the bond strength of conventional Bis-GMA-based resin to HF-etched zirconia and compare it to that of air-abraded zirconia. In order to focus on the micromechanical retention by etching, no primers were employed prior to the direct application of the resin to the conditioned zirconia ceramic. In addition, the surfaces etched with HF were characterized using various surface-analytical techniques. The experimental groups used in this study are summarized in Table 1.

**Table 1:** Experimental specimen groups and their different surface treatments.

Group Code	Surface Treatment Method	Etching Time
APA	Airborne-particle abrasion	Not applicable
10F5 and 10F30	10% hydrofluoric acid etching	5 and 30 min, respectively
20F5 and 20F30	20% hydrofluoric acid etching	5 and 30 min, respectively
30F5 and 30F30	30% hydrofluoric acid etching	5 and 30 min, respectively

## 2. Results and Discussion

### 2.1. Surface Characteristics

#### 2.1.1. Scanning Electron Microscopy

**Figure 1:**
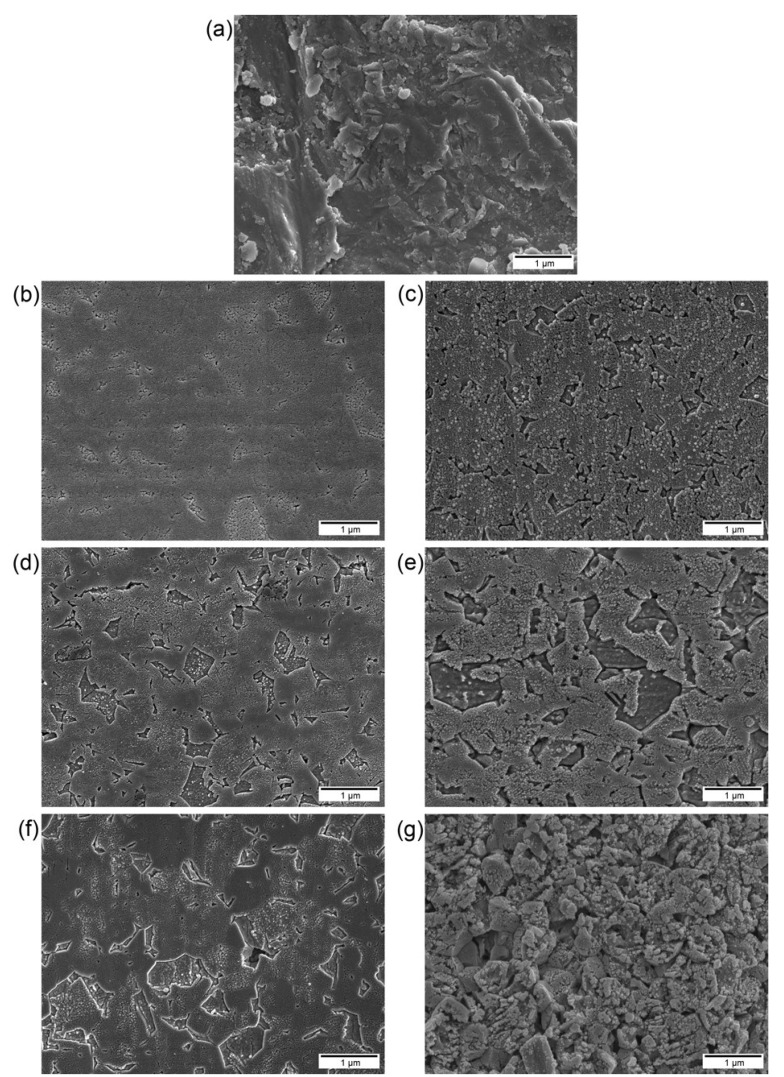
Scanning electron microscopy (SEM) surface images of the zirconia ceramic specimens after different surface treatments (20,000× magnification, bar = 1 μm): (**a**) APA; (**b**) 10F5; (**c**) 10F30; (**d**) 20F5; (**e**) 20F30; (**f**) 30F5; (**g**) 30F30.

**Figure 2:**
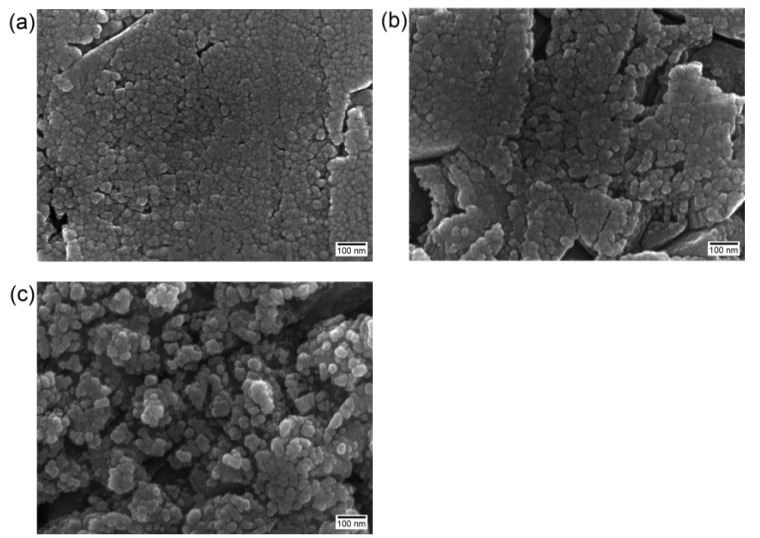
Scanning electron microscopy (SEM) images at high magnification showing nanoscale structures formed on 30-min etched surfaces (100,000× magnification, bar = 100 nm): (**a**) 10F30; (**b**) 20F30; (**c**) 30F30. Such nanostructures were not evident on the 5-min etched surfaces.

Representative scanning electron microscopy (SEM) images of the experimental groups are shown in Figure 1 and Figure 2. Figure 1a shows a typical air-abraded zirconia surface. In dental applications (e.g., the etching of porcelain veneers or the intraoral repair of fractured porcelain restorations), HF at concentrations of 4% to 10% is typically utilized [20]. For example, a dental manufacturer (Bisco Inc., Schaumburg, IL, USA) instructs that its 4% and 9.5% HF gel should be applied to porcelain surfaces for 5–6 min and 90 s, respectively. However, such low concentrations and short application times seem to be ineffective for the etching of zirconia ceramic [17]. Although the HF-etched specimen groups showed less rough surfaces than the airborne-particle abrasion (APA) group, the irregularities observed on the surfaces increased with increasing HF concentration and application time (Figure 1b–g). Higher HF concentrations and longer etching times resulted in the etching of the entire surfaces along with the formation of both micro- and nano-scale porosities. In particular, group 30F30 showed definite emergence of nanoscale structures with high-density spherical protuberances on the surface, which were more evident at higher magnification (Figure 2c).

#### 2.1.2. Atomic Force Microscopy

**Figure 3:**
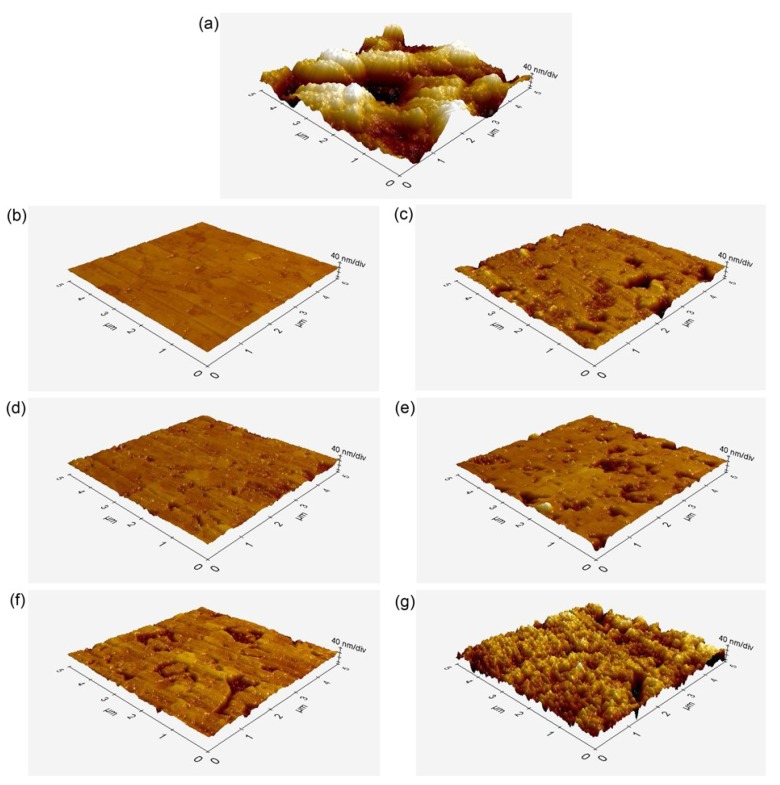
Atomic force microscopy (AFM) images of the zirconia ceramic specimens after different surface treatments (5 μm × 5 μm): (**a**) APA; (**b**) 10F5; (**c**) 10F30; (**d**) 20F5; (**e**) 20F30; (**f**) 30F5; (**g**) 30F30. Note that more retentive surface morphologies were produced with increasing concentration and application time of the etching solutions.

Figure 3 and Table 2 present representative atomic force microscopy (AFM) images and the surface roughness values calculated from these images, respectively. Figure 3a shows the AFM image of a zirconia surface aggressively roughened by air-abrasion. For air-abrasion of zirconia with Al_2_O_3_, various particle sizes and pressures have been employed. In this study, 110-µm Al_2_O_3_ particles at a pressure of 0.25 MPa were used. These conditions may be too aggressive and may damage the surface and induce structural defects [9,16]. Kern *et al.* [14] and Yang *et al.* [21] showed that such surface defects can be minimized by using smaller particles (e.g., 50 µm) and/or reducing the blasting pressure (e.g., 0.05 MPa) without affecting the long-term resin–zirconia bond strength. Differences in the zirconia surface texture were evident according to the different surface-treatment methods performed. The air-abraded surface showed a significantly higher *R*_a_ value than the HF-etched surfaces (*p* < 0.001). The AFM images also showed that the HF solutions roughened the zirconia surfaces in a substantially different way than did air-abrasion. The HF solutions partially dissolved the zirconia superficial grain structure (within as well as between the grains), resulting in high surface roughness at the nanometer scale, dependent on the concentration and application time. Within the HF-etched specimen groups, the mean *R*_a_ values increased with increasing concentration and application time. *R*_pv_ showed a similar tendency like *R*_a_. However, the formation of nanoscale structures was not evident after 5 min of etching. This finding indicates that zirconia etching by HF proceeds only slowly, even when HF solutions of high concentration are used [22].

**Table 2:** Average surface roughness *R*_a_ and peak-to-valley roughness *R*_pv_ of the zirconia ceramic specimens (mean (standard deviation) in nm).

Group	*R*_a_	*R*_pv_
APA	106.14 (17.07) a	485.05 (85.30) a
10F5	2.70 (0.62) b	19.53 (3.19) b
10F30	9.72 (1.06) cd	71.61 (12.88) c
20F5	7.58 (1.49) c	51.23 (14.74) c
20F30	13.07 (2.81) d	74.29 (12.79) c
30F5	10.51 (1.40) d	65.51 (11.93) c
30F30	30.73 (4.74) e	179.98 (32.69) d

Means with the same lowercase letter are not statistically different (*p* > 0.05).

#### 2.1.3. Water Contact Angles

**Figure 4:**
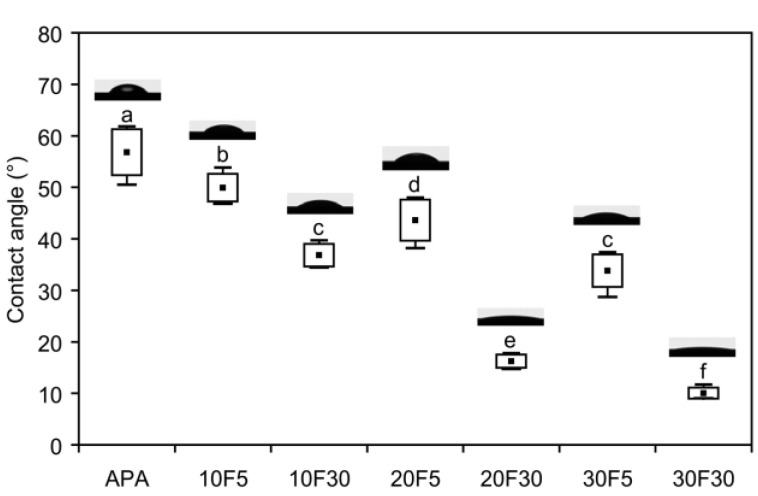
Contact angles of water droplets on the specimen surfaces. Black squares denote mean values, boxes represent standard deviations, and whiskers define the minimum and maximum values. Identical lower-case letters indicate statistically equivalent values (*p* > 0.05).

The water contact angle (CA) data are shown in Figure 4. It is known that larger roughness improves wetting for CA θ < 90° but enhances hydrophobicity for θ > 90° [23,24]. The HF-etched specimens showed significantly lower CAs than the APA group (*p* < 0.05). Within the HF-etched specimen groups, the *R*_a_ values increased (Table 2) and the CA values decreased (down to 10.0°) with increasing HF concentration and application time of the etching solutions. Moreover, for each HF concentration, longer application time (30 min) resulted in significantly higher *R*_a_ and significantly lower CA values.

#### 2.1.4. X-ray Photoelectron Spectroscopy

**Figure 5:**
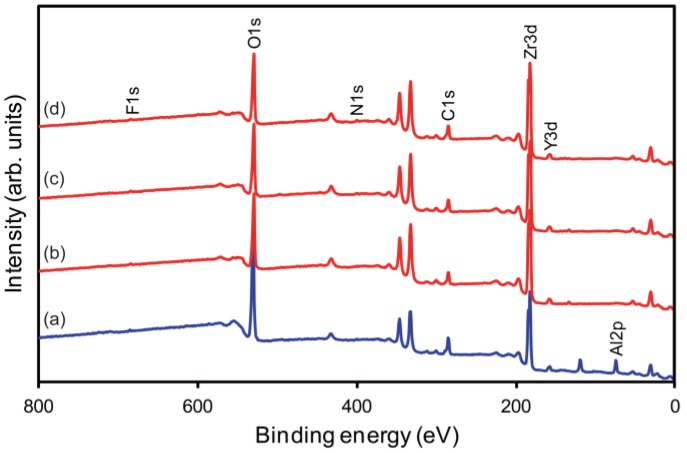
Wide-scan X-ray photoelectron spectroscopy (XPS) spectra in the range from 0 to 800 eV: (**a**) APA; (**b**) 10F30; (**c**) 20F30; (**d**) 30F30. Al: aluminum; C: carbon; F: fluorine; N: nitrogen; O: oxygen; Y: yttrium; Zr: zirconium.

Figure 5 shows the wide-scan X-ray photoelectron spectroscopy (XPS) spectra, in which the peaks were calibrated *vs.* the C1s peak of the hydrocarbon species at 285 eV. Aluminum at the level of 12.7 at% was detected in group APA. Fluorine at the concentration of 0.4 at% was consistently detected on the surfaces, regardless of the concentration of the HF solution used. Thus, among the HF-etched specimen groups, the differences in CA (Figure 4) seem to arise mainly from differences in surface topography [25].

#### 2.1.5. Fourier-Transform Infrared Spectroscopy

**Figure 6:**
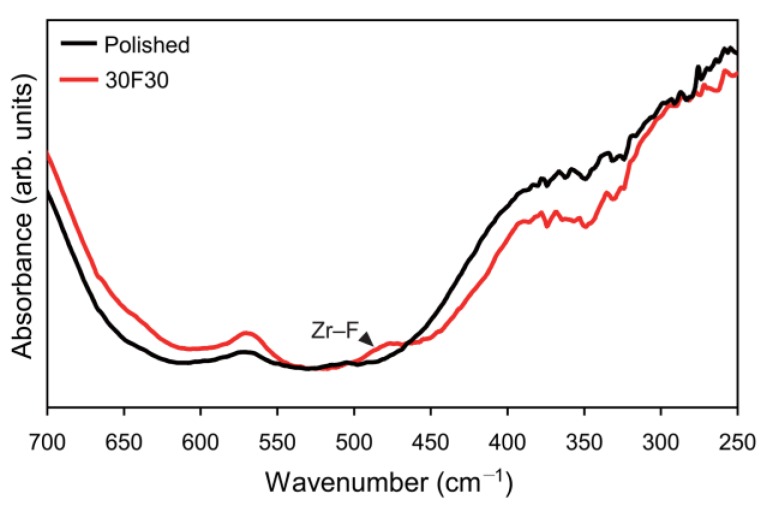
Far-infrared (FIR) spectra of zirconia polished and etched with 30% HF for 30 min (30F30).

The far-infrared (FIR) spectra of the polished and the 30F30 specimens are shown in Figure 6. The characteristic IR band assigned to asymmetric stretching of both bridging and non-bridging Zr–F bonds appeared at 518–488 cm^−1^. Thus, it seems that a small amount of fluorine, which remained after post-etching cleaning, formed Zr–F bonds at the zirconia surface [26].

### 2.2. In Vitro Fluoride Release

**Figure 7:**
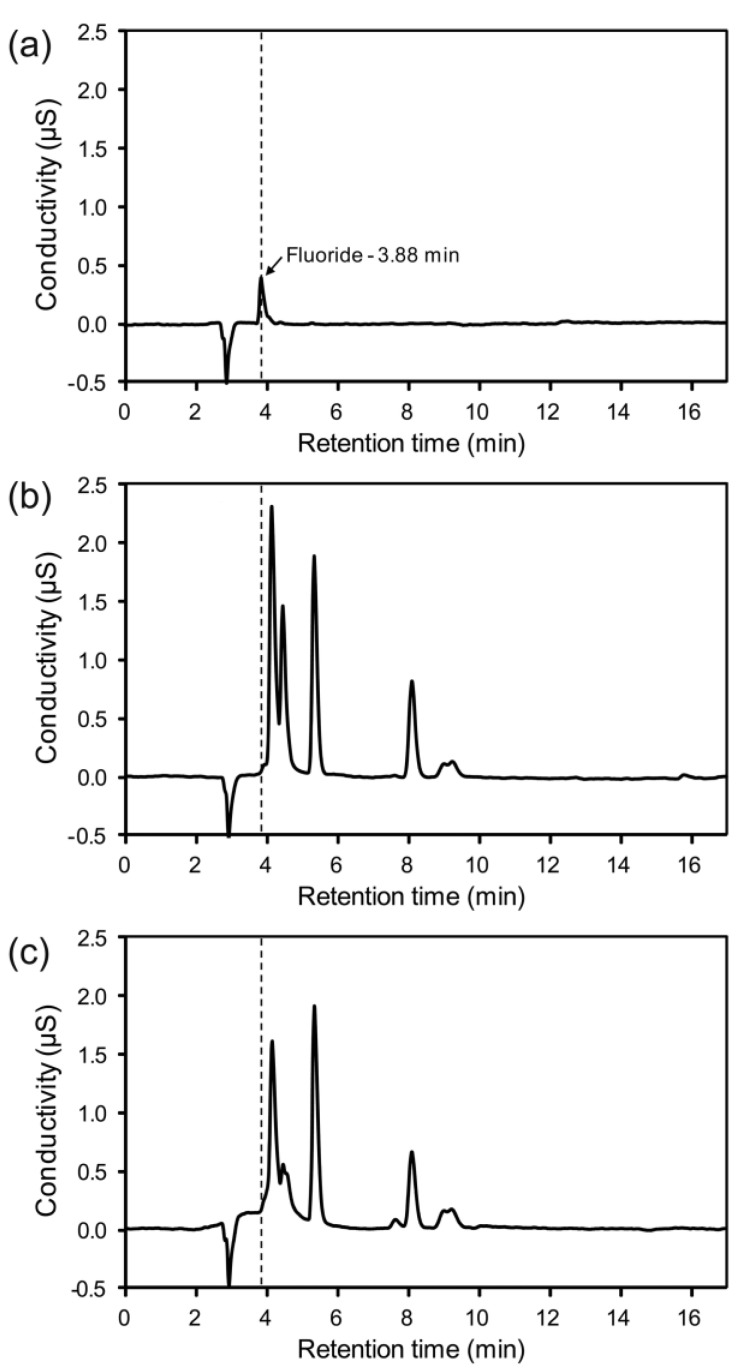
Ion chromatograms of (**a**) 0.1 ppm fluoride standard solution; (**b**) five-day storage water of the 30F30 specimen; and (**c**) five-day storage water of the resin (Duo-Link)-covered 30F30 specimen. Note that no distinct peaks corresponding to fluoride appeared at the characteristic retention time of 3.88 min in the specimens.

Figure 7 shows the ion chromatograms of 0.1 ppm fluoride standard solution and of the storage water of the tested zirconia specimens. Calibration data of fluoride showed good correlation between peak area and concentration (*r* > 0.999). No peaks corresponding to fluoride were detected at 0.1 ppm resolution over the entire experimental period of 30 days, indicating that fluoride ions were not significantly released from the specimen into water. Previous studies demonstrated that fluorination of zirconia converts the surface to more reactive zirconium oxyfluoride (ZrO*_x_*F*_y_*), thereby improving the chemical bonding with the resin composite [5,27]. Similarly, the fluoride phase formed on the HF-etched zirconia surface could modify the surface to become more reactive than the non-etched surface. Although the cross-linking resin monomers contained in non-adhesive resins, such as Duo-Link, are rather hydrophobic, they also have hydrophilic functional groups (e.g., hydroxyl) [28]. Thus, it is possible that the fluoride phase chemically reacts with such hydrophilic functional groups of the resin [29]. However, further investigations are still required to verify this assumption.

### 2.3. Shear Bond Strength and Failure Pattern

The means and standard deviations of the shear bond strengths before and after thermocycling are summarized in Table 3. In general, most approaches for establishing a resin–zirconia bonding are based on the formation of mechanical bonding or chemical adhesion, or a combination of these two mechanisms [27]. It should be noted that the “non-self-adhesive” (Bis-GMA-based) resin cement Duo-Link was directly applied to air-abraded or HF-etched zirconia ceramic surfaces [2]. Thus, the bonding mechanism seems to be primarily based on mechanical interlocking by the polymerization of the low-viscosity resin infiltrated into the roughened zirconia ceramic surfaces [17,30].

**Table 3:** Resin shear bond strength of the zirconia ceramic specimens subjected to different surface treatments before and after thermocycling (mean (standard deviation) in MPa).

Group	24 h-Water Storage	5000 Thermocycling
APA	6.6 (1.1) Aa	3.7 (0.6) Ab
10F5	8.3 (1.2) Aa	4.9 (0.8) Bb
10F30	13.5 (1.6) Ba	11.5 (1.8) CDb
20F5	12.6 (2.8) Ba	9.6 (2.1) Cb
20F30	25.2 (3.0) Ca	21.0 (3.0) Eb
30F5	14.9 (2.7) Ba	12.7 (1.6) Db
30F30	34.7 (4.1) Da	29.8 (3.9) Fb

Within the same column, means with the same uppercase letter are not statistically different (*p* > 0.05). Within the same row, means with the same lowercase letter are not statistically different (*p* > 0.05).

Previous studies have shown that air-abrasion with Al_2_O_3_ particles is an essential step in achieving a durable bond to zirconia ceramics [15,19,30,31,32]. Before thermocycling (24-h water storage), group APA showed significantly lower shear bond strength than all groups of HF-etched specimens (*p* < 0.001), except for group 10F5 (*p* = 0.137). The AFM image (Figure 3a) and high CA value (56.8°, Figure 4) of group APA also suggest that high surface roughness values do not necessarily indicate the formation of an optimal retentive surface topography for mechanical bonding [17]. This is in agreement with previous studies showing that conventional Bis-GMA-based resin materials without adhesive monomers were unable to create durable bonds to air-abraded zirconia [14,15,19,33].

For a given HF concentration, a longer application time (30 min) significantly increased the bond strength compared with a shorter one (5 min). In SEM observations, group 10F5 exhibited a relatively smooth surface with few signs of retentive sites formed (Figure 1b). The shear bond-strength value of group 10F5 may be considered to be too low to ensure good clinical service [23,33]. The other HF-etched specimen groups, which contained more retentive surfaces, produced clinically acceptable initial shear bond strengths (*i.e.*, greater than 10 MPa). Moreover, all etched specimen groups, except for group 10F5, showed relatively durable bond-strength values after thermocycling (up to 29.8 MPa), as well as high initial values. Both before and after thermocycling, group 30F30 achieved the highest bond strength among all investigated groups (*p* < 0.001).

Thermocycling and water storage are the popular methods used for artificially ageing the bonded specimens, thereby testing the durability of adhesion [34,35]. Thermocycling combines the hydrolytic effect and thermal stresses, and therefore, may simulate the natural process of ageing of the bonded interface. In this study, the durability of resin–zirconia bonding was evaluated using 5000 thermocycling, in accordance with ISO 10477 [36]. The Bis-GMA-based resin used did not contain adhesive monomers and no adhesive primers were employed prior to the application of the resin. The difference between the linear coefficients of thermal expansion (LCTEs) is particularly high between zirconia and resin cements, which have lower filler content than conventional restorative composite resins [35]. Thus, the significant decreases in bond strength after thermocycling (Table 3) may be mainly attributable to thermal stresses developed at the resin–zirconia interface because of the difference in LCTE of resin and zirconia, rather than hydrolysis of the resin during thermocycling [34,35].

However, thermocycling has been criticized regarding the extent to which it mimics clinical situations. The prescription of ISO 10477, namely 5000 thermocycles [36], may be insufficient for evaluating the long-term stability [35]. Thus, the thermocycling test results (Table 3) should be considered with caution because there is no definite evidence that failure occurred mainly because of thermal stresses [35]. Water storage could be regarded as the preferred method to age resin–zirconia bonds in the assessment of bond durability [37]. Kern and Wegner [30] used short-term water storage and compared it to long-term water storage combined with 37,500 thermocycles. Thus, extended cycling along with long-term water storage at 37 °C are more appropriate to evaluate the long-term stability of resin–zirconia bonds [14,21,30,35,37].

All debonded specimens showed adhesive failure at 10× magnification, regardless of the experimental group or thermocycling conditions. However, such adhesive failure did not indicate poor resin–zirconia bonding because even the groups that produced high bond-strength values showed adhesive failure.

### 2.4. Cross-Sectional Images

**Figure 8:**
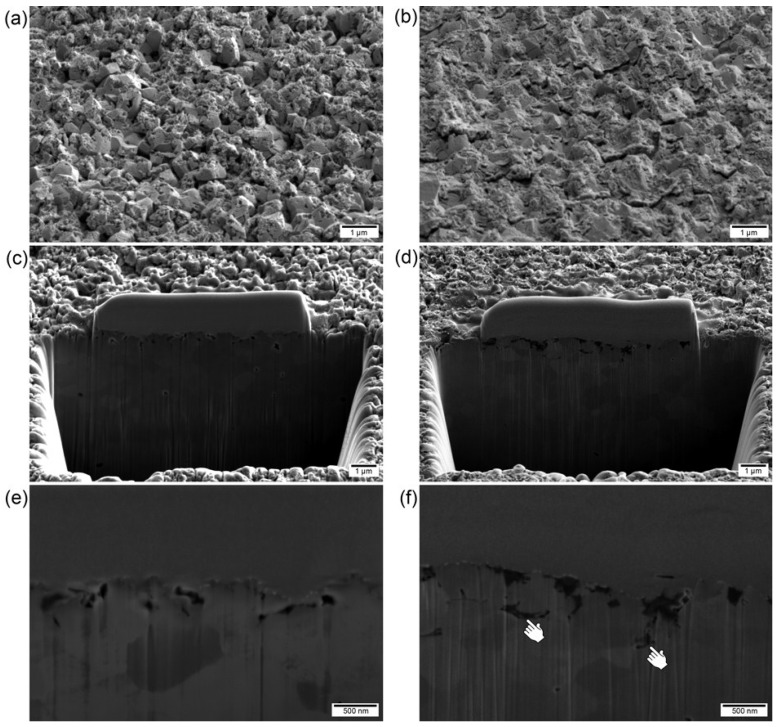
Scanning electron micrographs (SEM) of 30F30 specimens etched (left) and debonded (right): (**a**,**b**) surface images (20,000× magnification, bar = 1 μm), (**c**,**d**) overview of cross-sections prepared by FIB (15,000× magnification, bar = 1 μm), (**e**,**f**) enlarged cross-sections (50,000× magnification, bar = 500 nm). In Figure 8f, the pointers indicate incomplete resin infiltration into the micropores.

The etched and adhesively debonded surfaces of a specimen of group 30F30 were compared after the preparation of cross-sections by focused ion beam (FIB) (Figure 8). Unlike the etched surface, the debonded surfaces showed that some of the low-viscosity resin penetrated the shallow micro- and nano-pores and later fractured at narrow necks of resin tags. Thus, such a shallow etched surface architecture may be desirable in that it allows infiltration of the low-viscosity resin into the created retentive features and, at the same time, does not result in deep and excessive surface damage or exaggerated surface roughness which could weaken the treated zirconia [16]. In a recent study, however, HF etching could induce the tetragonal-to-monoclinic phase transformation of zirconia due to low-temperature degradation [38]. Thus, further investigations are needed to confirm the mechanical and physical properties of HF-etched dental zirconia. Figure 8f shows that the microporosities were not completely filled with the low-viscosity resin Duo-Link, despite a higher bond-strength value. The significant decrease in bond strength in all etched groups after thermocycling may be attributed to the incomplete infiltration of the resin cement into the micropores. In addition, the relatively low bond-strength values for all groups after thermocycling (Table 3), when compared to those of some previous studies [14,21], may indicate the lack of additional chemical adhesion between resin and zirconia. Thus, more durable bonding between resin composite and the HF-etched zirconia ceramic could be achieved by the use of resin cement containing hydrophilic functional monomers or by the pre-treatment of hydrophilic primers prior to the application of Bis-GMA-based resin cement [10,12,14,21,39].

## 3. Experimental Section

### 3.1. Etching Solutions and Zirconia Specimens

HF was purchased from Sigma-Aldrich Co. (St. Louis, MO, USA; lot #: MKBH5499V). Using this acid, experimental HF etching solutions with three different concentrations of 10, 20, and 30 wt% (codes: 10F, 20F, and 30F, respectively) were produced.

Rectangular (10 × 10 × 1 mm^3^) zirconia ceramic plates (Katana system, Noritake Dental Supply Co., Ltd., Miyoshi, Japan) were prepared according to the manufacturer’s instructions. One surface of each specimen was polished with silicon carbide (SiC) paper up to #2000 and, finally, with nylon cloths using diamond pastes of 1 and 0.5 μm grit size [17]. Specimens were ultrasonically cleaned in isopropyl alcohol and in distilled water for 15 min each, air-dried [23], and divided into seven groups according to the following surface treatment methods (Table 1): air-abrasion with 110-µm Al_2_O_3_ particles from a distance of 10 mm perpendicular to the specimen surface at a pressure of 0.25 MPa for 13 s (control group) [23]; 10F, 20F, or 30F etching either for 5 or 30 min at room temperature (23 ± 1 °C) and relative humidity of 50% ± 5% [40]. The treated specimens were then washed with running distilled water for 1 min, ultrasonically cleaned in acetone, in ethanol, and in distilled water for 15 min each, and finally air-dried.

### 3.2. Surface Characterization

#### 3.2.1. Scanning Electron Microscopy

One representative specimen from each group was prepared for field emission scanning electron microscopy (FE-SEM, JSM-6700F, Jeol, Tokyo, Japan). The specimens were sputter-coated with platinum, and photographs of representative areas of the surfaces were taken.

#### 3.2.2. Atomic Force Microscopy

Three zirconia specimens of each group were prepared for atomic force microscopy (AFM, XE-100, Park Systems Corp., Suwon, Korea). During the analysis, the microscope was operated in non-contact mode and a Si_3_N_4_ V-shaped cantilever (*k* = 42 N/m) was used. Images with 256 × 256 pixels were taken in air with scan size of 5 µm × 5 µm and at a scan rate of 0.5 Hz. Using the AFM images, the average surface roughness *R*_a_ and the peak-to-valley roughness *R*_pv_ were calculated [41]. Three measurements were taken for each specimen using a standardized rectangular spot (1.5 µm × 1.5 µm).

#### 3.2.3. Water Contact Angles

To compare the surface wettability, the contact angles (CAs) of water droplets on the zirconia surfaces were measured using the static sessile-drop method by a surface goniometer (OCA 15 plus, Data-Physics Instrument GmbH, Filderstadt, Germany). Five specimens per group were prepared for the CA measurements. All measurements were performed in a temperature-controlled room at 23 ± 1 °C with relative humidity of 50% ± 5% [40].

#### 3.2.4. X-ray Photoelectron Spectroscopy

X-ray photoelectron spectroscopy (XPS) was used to identify possibly existing residual elements on the zirconia surfaces. All measurements were performed using an XPS system (PHI Quantera SXM, ULVAC-PHI Inc., Tokyo, Japan) equipped with an X-ray source providing Al Kα radiation with an energy of 1486.6 eV. The emission angle of the photoelectrons was kept constant at 45°. A 180° hemispherical analyzer with 32-channel detectors was used for the detection of the photoelectrons [13]. Wide-scan survey spectra covering the range of 0–1350 eV were recorded to examine the surface composition of the specimens under ultrahigh vacuum at 10^−7^ Pa [13].

#### 3.2.5. Fourier-Transform Infrared Spectroscopy

The surfaces of a polished and a 30F30 specimen were analyzed using a Fourier-transform infrared (FTIR) spectrophotometer (IRTracer-100, Shimadzu Corp., Kyoto, Japan) equipped with diffuse reflectance accessory (DRS-8000, Shimadzu Corp.). Far-infrared (FIR) spectra were collected over the range of 700–250 cm^−1^ at a resolution of 4 cm^−1^ by performing 20 scans. The spectral correction was performed employing the Kubelka–Munk algorithm [26].

### 3.3. Ion Chromatography

Fluoride release from the 30F30 specimens in distilled water was tested by ion chromatography (IC). For each zirconia specimen, all surfaces except one (10 × 10 mm^2^) were covered with a composite resin (Aeliteflo, Bisco Inc.), which was then light-cured using a quartz-tungsten-halogen curing light (Elipar TriLight, 3M ESPE; standard mode, output intensity = 750 mW/cm^2^). After immersion in F30 etching solution for 30 min, the zirconia specimens were removed from the covering resin and cleaned as described above. A total of six zirconia specimens were prepared. For half of them, freshly mixed dental resin cement (Duo-Link, Bisco Inc., lot #: 1300003751) covered the entire etched surfaces with a thickness of 1 mm and was light-cured for 40 s using the Elipar TriLight curing light. This light-curing was performed from each side of the resin cement to ensure optimal polymerization. All specimens were immersed and stored in individual plastic containers filled with 5 mL of distilled water at 37 °C for 5 days. After that, each specimen was removed from its container and placed in a new one, again filled with 5 mL of distilled water (37 °C). This procedure was repeated every 5 days for 30 days in total (5, 10, 15, 20, 25, and 30 days).

The fluoride concentration of the water samples was measured by IC (ICS-5000, Dionex, Sunnyvale, CA, USA) with a resolution of 0.1 ppm (using fluoride calibration standard solutions of 0.1, 0.5, 1.0, and 10 ppm). As mobile phase, 20 mM potassium hydroxide was used. The instrument was fitted with an IonPac AS19 analytical column and IonPac AG19 guard column. The injection volume was 25 µL and the flow rate was 1.0 mL/min. Free fluoride ions have a well-defined retention time and the peak corresponding to fluoride could readily be determined from the chromatogram [42].

### 3.4. Shear Bond-Strength Testing

Fifty-six sintered zirconia specimens were embedded in round silicone-rubber molds using an acrylic resin and their exposed surfaces were treated as described above. The zirconia surfaces to be bonded were isolated using a bonding jig (Ultradent Products Inc., South Jordan, UT, USA). The freshly mixed resin cement (Duo-Link) was applied to the surface by packing the material into cylindrically shaped plastic matrices (Ultradent Products Inc.) with an internal diameter of 2.38 mm [24] and then irradiated for 40 s using the Elipar TriLight curing light. In this manner, three bonded resin cylinders were made on one treated zirconia surface (*n* = 12/group). All bonded specimens were stored in distilled water at 37 °C for 24 h. Half of them were additionally thermocycled 5000 times between 5 and 55 °C in water baths with a dwelling time of 30 s and an exchange time of 5 s between each bath, according to ISO 10477 [36].

The specimens were then perpendicularly engaged at their bonded cement cylinder bases with a round-notched custom shear blade (Ultradent Products Inc.) in a universal testing machine (3343, Instron Inc., Canton, MA, USA). Shear bond-strength testing was performed at a crosshead speed of 1.0 mm/min until bonding failure occurred. The bond strengths (MPa) were calculated by dividing the peak load at failure (N) by the bonded surface area. Following debonding, all fractured interfaces were examined under an optical microscope (SMZ800, Nikon Corp., Tokyo, Japan) at 10× magnification to determine the mode of fracture according to one of the following three types: A, adhesive failure at the zirconia–resin cement interface; C, cohesive failure within the resin cement; and AC, a combination of these failure modes.

### 3.5. SEM Cross-Sectional Investigation

For group 30F30, cross-sectional images of the etched and debonded surfaces were examined using focused ion beam (FIB, Versa 3D LoVac, FEI Company, Eindhoven, The Netherlands) preparation and SEM investigations. The experimental protocols were described in detail elsewhere [43]. Briefly, the specimen was tilted by an angle of 52°, and observation of the cross-sections with the electron beam was also done at this angle (52°). Prior to the cross-sectional preparation, a platinum layer was deposited over only a small area at the point of interest to protect the surface against redeposition of ablated atoms. At predefined areas of interest, cross-sections were coarsely prepared with gallium ions accelerated at 30 kV and then polished at a lower acceleration voltage of 5 kV. The SEM images were taken in high-vacuum mode using an acceleration voltage of 5 kV.

### 3.6. Statistical Analysis

All data were examined for the normality of their distribution by the Shapiro–Wilk test and for the equality of the variances by the Levene test. The CA values of the different groups were compared using one-way ANOVA followed by Tukey’s *post hoc* test. For the roughness and bond-strength values, non-parametric statistical test procedures were used for group comparisons because the Leven’s test showed inhomogeneity of the variances between different groups (*p* < 0.001) [17]. The Kruskal–Wallis test was employed to compare different experimental groups, followed by the Mann–Whitney *post hoc* test, with adjustment of the significance level using the Sidak correction for multiple testing. Meanwhile, the Mann–Whitney test was used within each experimental group for the two thermocycling conditions of 0 and 5000 cycles. All statistical analyses were carried out using SPSS 17.0 for Windows (SPSS Inc., Chicago, IL, USA) at a level of significance of α = 0.05.

## 4. Conclusions

Within the limitations of this *in vitro* study, the HF-etching of zirconia ceramic surfaces may allow a low-viscosity Bis-GMA-based resin to penetrate and flow into the thus-formed micro- and nano-retentions, thereby creating a mechanical interlock after polymerization of the resin. The surface topography of HF-etched zirconia suggests that the application time is more critical than the concentration for the formation of retentive sites. The room temperature HF etching may have an advantage in comparison to other novel zirconia etching techniques that include a heat-treatment step. Nonetheless, it is not clear from this study whether using hazardous HF at high concentration and long exposition times in combination with Bis-GMA-based resin without adhesive monomers has a definite merit over low- or moderate-pressure air-abrasion in combination with phosphate monomer containing primers and/or luting resins. In addition, the mechanical properties of zirconia after HF etching should be further evaluated. Finally, long-term clinical investigations are necessary to ensure the clinical efficacy of HF etching of zirconia for improved resin–zirconia bonding.
